# Why school tobacco bans fail: staff engagement in enforcement in Belgian schools

**DOI:** 10.1093/heapro/daag031

**Published:** 2026-03-03

**Authors:** Pierre Laloux, Arja Rimpelä, Vincent Lorant

**Affiliations:** Institute of Health and Society (IRSS), Université catholique de Louvain, Clos Chapelle-aux-Champs 30, Brussels 1200, Belgium; Tampere University, Faculty of Social Sciences, Unit of Health Sciences, SOC, Tampere 33014, Finland; Tampere University Hospital, Department of Adolescent Psychiatry, The Wellbeing Services County of Pirkanmaa, PL 2000, Tampere 33521, Finland; Institute of Health and Society (IRSS), Université catholique de Louvain, Clos Chapelle-aux-Champs 30, Brussels 1200, Belgium

**Keywords:** smoke-free policies, implementation, school, staff, enforcement

## Abstract

Tobacco control bans are only as effective as their enforcement, and schools are a critical though often overlooked frontline. School tobacco policies (STPs) frequently fail when staff hesitate to act. This study aimed to measure and explain staff engagement in STP enforcement using a newly developed scale. Staff members (*n* = 624) from 18 Belgian secondary schools took part in the ADHAirE study. Building on previous qualitative studies, we constructed a 10-item scale to assess staff willingness to enforce the STP. We used exploratory factor analysis (EFA) to identify key attitudinal dimensions underlying the scale and conducted multilevel analyses to assess factors that predict enforcement. Participants scored high on willingness to enforce the rules (2.89, range 1–4). EFA revealed two key attitudinal dimensions, each explaining 20% of the variance: the perception that enforcement is a *professional role*, which was widely endorsed, and *sharedness of rules*, for which there was less adherence: only half of the participants felt supported by parents, and 40% believed tobacco control was not a school priority. Notably, enforcement was predicted by the perception that rules are enforced by one’s colleagues and the sense that the rules are justified. Bans are likely to be enforced if they are perceived as part of the staff’s professional role and are shared among the school community. Effective enforcement of school smoking bans requires a shared commitment that integrates school staff, leadership, and parents. The validated scale offers a tool for school principals or health authorities to assess that alignment.

Contribution to Health Promotion

- School tobacco bans are essential for preventing adolescent smoking, but their effectiveness depends on their effective implementation.
- To date, school staff perspectives on the enforcement of these bans have received little attention and have been investigated only through qualitative studies.
- This study shows that schools cannot expect to become smoke-free environments without the engagement of the school staff.
- Staff members will enforce the bans only if they feel supported and not isolated in their enforcement efforts.

## Introduction

Banning smoking from public places is recommended by the World Health Organization (WHO) to protect people from second-hand smoke, but it also influences people’s opportunities to smoke and is likely to de-normalize smoking (WHO 2008, 2009, Wilson *et al*. 2012). Bans are central to smoke-free policies, yet their effectiveness is strongly related to the capacity to enforce them (Gunningham 2010). School tobacco policies (STPs) are one strategy for preventing smoking. They regulate whether smoking is allowed in schools at all or where and when students/school staff are not allowed to smoke (Evans-Whipp *et al*. 2010). STPs appear more promising than an educational approach to smoking prevention because they target the social context in which young people are exposed to or can access tobacco products (Galanti *et al*. 2014). Nevertheless, evidence regarding their effectiveness in reducing adolescent smoking remains mixed (Galanti *et al*. 2014). Earlier studies did not necessarily take inconsistent or poor implementation into account but assumed a direct link between STPs and children’s behaviour. Inconsistent enforcement of STPs has been shown to undermine their effectiveness, as student-perceived enforcement is associated with decreased smoking on school premises (Lipperman-Kreda *et al*. 2009, Mélard *et al*. 2020). This study presents the development and validation of a novel instrument to measure the willingness of secondary school staff to enforce an STP and examine the factors underlying this willingness. We view school staff engagement in STP enforcement through the lens of the Advocacy Coalition Framework (AFC), which distinguishes between deep core, policy core, and secondary beliefs (Sabatier and Weible 2019). According to the theory, changing factual beliefs is one of the first steps in changing a norm and can be achieved by changing collective expectations (Bicchieri 2016, Sabatier and Weible 2019).

A qualitative study conducted among Dutch school staff members showed that core beliefs about smoking prevention in school settings may hinder STP enforcement (Schreuders *et al*. 2019): if staff members prioritize the values of individual freedom and autonomy over responsibility, perceive smoking as a secondary problem, fear for their positive relationship with students, or have doubts about the coherent enforcement of STPs (Schreuders *et al*. 2019). In their realist review, Linnansaari *et al*. (2019) identified three outcomes that must be achieved to facilitate STP enforcement (Linnansaari *et al*. 2019). First, staff must feel responsible because they experience the policy as part of their professional role and duties. Secondly, staff members should be motivated to enforce STPs because they perceive that their contribution leads to positive outcomes. Finally, feeling able to deal with students’ responses helps staff members to be confident about enforcing STPs. The situation regarding the enforcement of bans is peculiar to schools, as the enforcers and the subjects of the rules are socially close, which lowers willingness to enforce bans (Nolan 2017). The realist review is consistent with a Europe-wide qualitative study that highlighted the need for a supportive organizational structure, enforcement by the entire staff, and dialogue with students (Linnansaari *et al*. 2022). The latter is likely to be particularly important for staff to avoid interfering with already-vulnerable students who have a less supportive family background (Schreuders *et al*. 2020).

The effectiveness of STPs, therefore, depends on the willingness of staff to enforce them. As a smoke-free school cannot be achieved without the cooperation of staff members, understanding their willingness to enforce the STP and identifying the main arguments for and against it could guide interventions that address STP implementation. Since staff enforcement is crucial yet currently unmeasured, a scale is needed to assess and enhance enforcement efforts and thereby improve the overall effectiveness of STPs in reducing student smoking and vaping. To date, however, no quantitative measure has captured the willingness to enforce, leaving STPs a rather wishful strategy. This paper proposes a new measure for STP enforcement and identifies its main dimensions and key individual and school-level factors that influence enforcement.

## Methods

### Design and sample

This study is based on baseline data from the ADHAirE (A Decent and Healthy Air for Everyone) experimental study. Data were collected in secondary schools in the Hainaut province, Belgium. This province is known to have a high prevalence of smoking (22% current smokers vs. 19.4% in Belgium) despite smoking at school being prohibited since 2006. The study received ethical approval from the *Comité d’éthique hospitalo-facultaire UCLouvain* (reference number 2023/30OCT/438). Further details of the methodological features of the ADHAirE study can be found elsewhere (Laloux *et al*. 2025). The study follows STROBE standards of reporting (Additional file 1).

Data collection took place in 18 secondary schools between October and December 2024. In total, 624 staff members out of 2031 participated (response rate: 31%). Consent to participate was obtained before the completion of the questionnaire. They completed a self-reported online survey in which they were asked about their smoking behaviour, their perceptions of the STP, and sociodemographic factors. For complementary analysis, we also used data collected on students in the third and fourth grades from those schools. In total, 3064 adolescents out of 4347 took part in the survey (response rate: 70%). They completed an online survey in class in which they reported their smoking and vaping status, their perceptions of the STP and its enforcement, and sociodemographic information. Here again, informed consent to participate was obtained before the completion of the questionnaire. In Belgium, parental consent is not required for survey participation among children aged 13 years and older.

### Measurements

#### Enforcement

Based on the theoretical background provided by Linnansaari *et al*. (2019), we developed a scale to assess staff willingness to enforce an STP, proposing three mechanisms (Linnansaari *et al*. 2019):

- Mechanism 1: When contextual factors cause school staff to experience the STP as part of their professional role and duties, it can lead to staff members taking responsibility for STP enforcement.
- Mechanism 2: When contextual factors cause staff members to perceive their contribution as leading to positive outcomes, it may lead to staff members showing motivation for STP enforcement.
- Mechanism 3: When contextual factors cause staff to feel able to deal with students’ responses, it may lead to staff members showing confidence in STP enforcement.

Based on this framework, a preliminary list of items was selected by PL. The items measure staff perceptions of STP enforcement that influence willingness to enforce an STP. The list was then discussed with an expert in STPs (V.L.). A first version was piloted, with cognitive testing to ensure clarity and relevance. Following feedback from the staff members of a Brussels school who tested the questionnaire, we clarified the phrasing and kept the items for which we found the best theoretical alignment to the framework’s dimensions. The second version was then also tested for clarity (see scale building progress in Additional file 2). Table 1 presents the 10 items, related to the three mechanisms, which were retained and which the staff members were questioned about. Each item is rated on a four-point scale, ranging from ‘I totally disagree’ (1) to ‘I totally agree’ (4). In the distribution analysis, agreement was defined as selecting either option 3 or 4 on the scale.

**Table 1:** Items of staff members’ willingness to enforce the STP, ADHAirE study 2024.

Mechanisms (9)	Questions (ADHAirE study)	Proportion of agreement (%, 95% CI)
Mechanism 1: responsibility is part of professional role and duties	1.1. It is my responsibility to enforce these rules	88.6 (86.0–91.2)
	1.2. I feel I have legitimacy to enforce these rules	83.0 (79.9–86.1)
	1.3. Smoking prevention is a priority for my school	61.9 (57.9–65.9)
Mechanism 2: staff contribution to positive outcomes to motivate enforcement	2.1. These rules help protect students from smoking and vaping	73.1 (69.5–76.7)
	2.2. I feel supported by my colleagues to enforce these rules	70.7 (67.0–74.4)
	2.3. Students always get around these rules	60.8 (56.8–64.8)
	2.4. I feel supported by the parents to enforce these rules	52.2 (48.1–56.3)
Mechanism 3: staff’s ability to enforce the rules to be confident to enforce the STP	3.1. I can enforce these rules while remaining close with my students	92.4 (90.2–94.6)
	3.2. I know these rules well	64.0 (60.1–67.9)
	3.3. I risk damaging my relationship with my students if I enforce these rules	14.7 (11.8–17.6)
Mean score (SD, range 1–4)	–	2.89 (0.49)

#### Explanatory factors of enforcement

In order to identify factors influencing staff willingness to enforce the STP, we considered information at both the school and individual levels. At the school level, we collected three indicators: the school’s socioeconomic status (SES), the visibility of smoking, and smoking initiation. The latter two indicators were derived from the students’ survey. Every school in the Fédération Wallonie-Bruxelles (FWB) gets an official school SES index score based on several factors, including parents’ income, employment status, and education level. Schools are then ranked on a scale from 1 (lowest school SES) to 20 (highest school SES). To assess the visibility of smoking, students were asked how often they saw other students smoking at the school entrance. They answered on a four-point scale, which ranged from ‘Never or very rarely’ (1) to ‘Every day or almost every day’ (4). For the analysis, we measured, for each school, the proportion of students who see other students smoking at the school entrance at least once a week (responses 3 and 4). Smoking initiation was measured, for each school, as the proportion of students who reported having tried smoking at least once. We chose ‘ever smoker’ rather than ‘current smoker’ as an indicator for two reasons. First, current smoking is still rare at that age (7.88% of our sample), which limits variability. Secondly, current smoking was found to have lower variance (0.07) than ever smoking (0.19). We therefore prioritized ever smoking to ensure greater variability in the data and improve the statistical power of the analyses.

At the individual level, we collected four indicators from the school staff members: smoking status and three perceptions regarding STP enforcement. To assess staff members’ smoking status, they could report that they were either a ‘never smoker’, ‘former smoker’, ‘occasional smoker’, or ‘daily smoker’. For the first perception regarding STP enforcement, school staff were asked, ‘Do you think that sanctions against students caught smoking/vaping are justified?’ to assess their attitude towards the STP. Answers ranged from ‘I totally disagree’ (1) to ‘I totally agree’ (5) on a five-point scale. For the second perception regarding STP enforcement, we asked, ‘In your school, are rules about smoking and vaping generally enforced?’ to assess the actual enforcement of the STP. Answers ranged on a four-point scale from ‘Totally disagree’ (1) to ‘Totally agree’ (4). For the last perception regarding STP enforcement, staff members were asked, ‘How do the staff react when a student is caught smoking or vaping?’. They could answer either ‘Each member of staff applies the rules in their own way’ (1), ‘Some members of staff apply the rules in the same way’ (2), or ‘Most staff members apply the rules in the same way’ (3). This perception reflects the consistency in enforcement and the wider school communication regarding the tobacco policy.

### Analysis

A missing data analysis was performed prior to the main analyses. The MCAR test (*P* = 0.506) indicated no systematic pattern of missingness. No significant differences were found between the complete-case sample and the full sample regarding age, sex, school role, or attitudes to smoking prevention. We therefore conducted a complete-case analysis (*n* = 577).

#### Assessment of the scale’s dimensions

To assess the scale’s properties in relation to the theoretical framework, we performed an exploratory factor analysis (EFA). Before analysis, the Kaiser-Meyer-Olkin (KMO) index value was calculated to measure the appropriateness of the data for factor analysis (Kaiser and Rice 1974). Values above 0.50 are considered suitable for factor analysis (Williams *et al*. 2010). We then calculated Cronbach’s alpha for the global questionnaire and for each item separately to assess internal reliability (Cronbach 1951). Values between 0.70 and 0.95 are considered acceptable (Tavakol and Dennick 2011). For the EFA, we used an oblique rotation, as orthogonality in the domain of enforcement behaviours is unrealistic (Williams *et al*. 2010). Factor extraction was performed following the principal components analysis, using Kaiser’s criteria and the Scree test as extraction criteria.

#### Enforcement predictors

To identify individual- and school-level predictors of willingness to enforce the STP, we used multilevel models, since the main outcome, willingness to enforce the STP, may be influenced by the school context. The dependent variables were the factors identified in the EFA. The score for each factor was calculated by taking the mean of the related items of the enforcement scale. Negative items (2.3. and 3.3.; see Table 1) were reversed. Predictors were used as explanatory variables. We first used an empty model (Model 0) to calculate the intra-class correlation (ICC) coefficient and evaluate the relevance of the multilevel analysis. We then performed two models (Models 1 and 2) related to the factors extracted in the EFA.

All statistical analyses were conducted in R Studio (version 4.4.1). The *psych* package (Revelle 2017) was used for the EFA and the *lme4* package (Bates *et al*. 2015) for the multilevel analysis.

## Results

### Sample characteristics

Participants were mainly women (63.7%) and were, on average, aged 44.2 years, with nearly 14 years’ seniority (13.8; Table 2), distributions close to that of school staff across the FWB (Wallonie-Bruxelles 2024). Nine out of 10 staff members (89.0%) were teachers, but educators (5.2%), principals (2.4%), and technical/administrative support staff (2.7%) also took part in the survey, to a lesser degree. In Belgium, a teacher’s mission is to teach and transmit knowledge and competences to students, whereas an educator’s role is to support students in their education and social lives, including with their well-being, personal issues, and discipline. Out of the staff members, 75.8% had a full-time position in the participating schools. The majority of the staff members (69.4%) had never smoked, 17.9% were former smokers, and 12.7% smoked regularly. Half of the schools had a SES score below 8.5, which is lower than the median of all schools in the FWB (SES median = 10). Results from the students’ survey show that the majority of them (61.8%) see other students smoking nearly every day at the school entrance. A quarter of them (25.5%) have tried smoking at least once.

**Table 2:** Characteristics of schools, staff members, and students, ADHAirE study 2024.

**School level (*n*** **=** **18)**	
Schools’ socioeconomic status (median, IQR; range = 1–20)	8.5 (4.8–11.0)
Schools’ average size (mean, SD)	
Number of students third–fourth grade	247.3 (112.1)
Number of staff	115.3 (39.9)
**Staff level (*n*** **=** **577)**	***N* (%)**
Age (mean, SD)	44.2 (9.7)
Sex (*n* = 557)	
Male	202 (36.3%)
Female	355 (63.7%)
Occupation type (*n* = 546)	
Principal	13 (2.4%)
Educator	28 (5.2%)
Teacher	486 (89.0%)
Technical/logistical/administrative staff	13 (2.7%)
Nursing staff	4 (0.7%)
Years of service (mean, SD)	13.8 (9.7)
Workload (*n* = 566)	
Full time	429 (75.8%)
Part time	137 (24.2%)
Smoking status (*n* = 576)	
Never smoker	400 (69.4%)
Former smoker	103 (17.9%)
Occasional smoker	31 (5.4%)
Daily smoker	42 (7.3%)

Students’ survey (*n* = 3064)	*N* (%)	School range [min–max]
Age (mean, SD)	15.1 (1.2)	[14.4–16.4]
Sex (*n* = 2860)		
Male	1554 (54.3%)	[25.0%–94.0%]
Female	1306 (45.7%)	[6.0%–75.0%]
Visibility of students smoking at the school entrance (*n* = 2922)		
Never of very rarely	342 (11.7%)	[22.2%–77.7%]
A few times a month	366 (12.5%)	[6.0%–18.9%]
Once a week	408 (14.0%)	[2.0%–17.4%]
Every day or almost every day	1806 (61.8%)	[10.7%–64.3%]
Tried smoking at least once (*n* = 3033)	773 (25.5%)	[6.3%–66.0%]

### Staff members’ willingness to enforce

Most staff members agreed with nearly all aspects of enforcement, for example, agreeing that they could enforce STPs while staying close to the students (Table 1; 92.4%), and a similarly high proportion felt responsible for enforcing the rules (88.56%). A minority of the staff members agreed with the statement that they risked damaging their relationship with the students by enforcing STPs (14.73%). Parental support for enforcement was found to have the lowest level of agreement, with only half (52.17%) of the participants feeling supported by parents to enforce the STPs.

### Dimensions of enforcement

**Figure 1:**
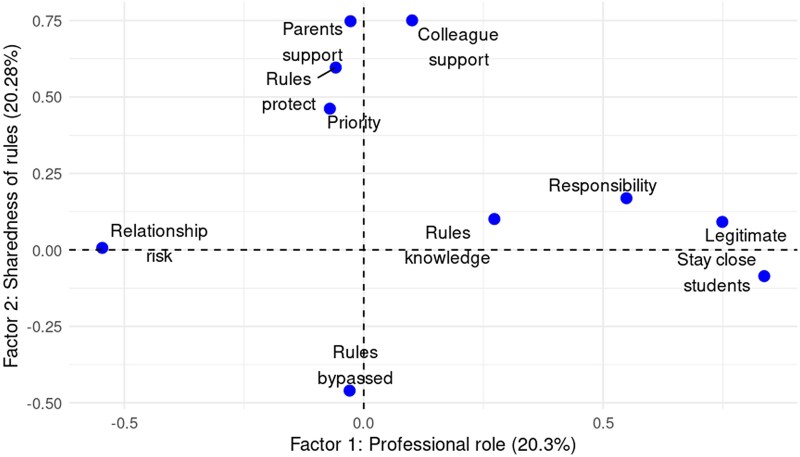
School staff willingness to enforce their STP, exploratory factor analysis (*n* = 577), ADHAirE study 2024.

The items showed good appropriateness for factor analysis (KMO index = 0.83) and good internal reliability (Cronbach’s alpha = 0.82). Following the Kaiser criterion, a solution was extracted that included two factors: professional role and sharedness of rules. This solution provided a more suitable structure in which factors correlated to multiple variables, unlike the three-factor model, in which one factor was linked to a single variable. Figure 1 shows the results of the EFA. Factor 1 accounted for 20.30% of the variance and Factor 2 for 20.28%. Factor 1 is mainly composed of items related to Mechanism 3 (confidence to enforce) and Mechanism 1 (responsibility to enforce): ‘Responsibility’, ‘Legitimacy’, ‘Stay close to students’, and ‘Relationship risk’. Factor 2 is mainly composed of Mechanism 2 items (motivation to enforce): ‘Parents’ support’, ‘Colleagues’ support’, ‘Rules protect’, ‘Priority’, and ‘Rules bypassed’. ‘Rules knowledge’ was dropped by the analysis because of too-low communality (0.13) and factor loadings. Detailed results of the EFA and preliminary analyses can be found in Additional file 2.

### Predictors of willingness to enforce

Table 3 displays the predictivity of enforcement. Nearly a third (ICC = 0.306) of the variance in staff members’ willingness to enforce the STP was attributable to the differences between schools. School-level factors were found to have no significant impact on the willingness of school staff to enforce the STP. Perceptions of enforcement (the sense that the rules are justified, the belief that the rules are most often enforced, and the perception of cohesive enforcement) were highly associated with both factors of willingness to enforce the STP. If staff scored one point higher (out of a maximum of four) on the scale measuring ‘Rules are often enforced’, they scored 0.11 points (out of a maximum of four) higher on the professional role factor. This suggests that when staff perceive that the rules are enforced at their school, they are more likely to feel responsible for and legitimate in enforcing the rules themselves. While being female was associated with a higher sense of professional role regarding STP enforcement, the association was not significant for the sharedness of rules factor. The staff members’ smoking status was found to have no significant association with either factor of willingness to enforce the STP, except for daily smoking status, which was negatively associated with the professional role.

**Table 3:** Predictors of school staff willingness to enforce the STP, multilevel analysis (*n* = 577), ADHAirE study 2024.

	Multilevel analysis b (95% CI)		
	Model 0	Model 1: factor 1 professional role	Model 2: factor 2 sharedness of rules
School random effect (ICC)	0.306	0.000	0.006
School-level factors			
School’s SES (low to high, 0–20)		0.00 (−0.01; 0.01)	0.01 (0.00; 0.02)
Visibility of smoking at the entrance		−0.02 (−0.20; 0.16)	−0.01 (−0.18; 0.15)
Smoking initiation		−0.57 (−1.36;0.21)	−0.42 (−1.09;0.29)
Individual-level factors			
Justification of the rules (disagree, to agree, score 1–5)		0.06 (0.01; 0.10)*	0.07 (0.03; 0.11)***
Rules are often enforced		0.11 (0.04; 0.18)**	0.26 (0.20; 0.32)***
Staff enforce rules cohesively		0.08 (0.01; 0.10)*	0.13 (0.07; 0.19)***
Smoking status			
Never		Ref.	Ref.
Former		−0.09 (−0.22; 0.03)	−0.01 (−0.12; 0.10)
Occasional		−0.13 (−0.33; 0.07)	0.01 (−0.17; 0.18)
Daily		−0.21 (−0.41; −0.01)*	−0.12 (−0.30; 0.05)

b (95% CI): coefficient estimate (95% confidence interval); *P*-value: <0.05*, <0.01**, <0.001***.

## Discussion

The success of school tobacco bans depends not only on the existence of rules but also on the school staff being committed to enforcing them, an aspect of implementation that, until now, has lacked a clear operational definition. This study addressed that gap by developing a reliable scale to measure staff willingness to enforce the STP.

This study emphasizes the essential role school staff members can play in smoking prevention by fostering a social environment that prohibits smoking. Traditionally, school smoking prevention has been viewed primarily as health education and is aimed directly at adolescents to alter their knowledge and skills (Thomas *et al*. 2013). Health education alone, however, may be inadequate for schools striving to create smoke- and nicotine-free environments, as it addresses individual behaviour in response to social influences (Mead *et al*. 2014).

The perspectives of community members on this issue are also crucial, as they can either support or undermine the smoke-free school policy (STP). Considering the viewpoints of staff members can enhance their support and acceptance of the STP (Kreuter *et al*. 2000). By identifying the facilitators and barriers to implementation through the insights of those involved in the intervention, schools can significantly improve the likelihood of successful policy execution (Pearson *et al*. 2015, Wolfenden *et al*. 2022).

### Main findings

Among school staff, the enforcement of school tobacco bans is shaped by two dimensions: *professional role* and *sharedness of rules*. The first dimension regards how the staff extends its professional role to tobacco rules while fostering a positive student–staff relationship. The second dimension concerns the sense of shared responsibility for the rules among the staff members and the wider educational community (e.g. parents). It is built on the sense that smoking prevention is an important task for all, the need for support, and the feeling of effectiveness when enforcing STPs. In general, most staff members felt that they were responsible and had the legitimacy to enforce the STP while maintaining a positive relationship with the students. A substantial proportion, however, did not feel supported by parents or by their colleagues. Furthermore, smoking prevention turned out not to be a priority for a large proportion of participants, and a significant share of them did not consider it effective. Staff members’ perceptions of how the rules were enforced by the rest of the educational community predicted their willingness to enforce the STP. Except for daily smokers, who do not perceive STP enforcement as part of their professional role, smoking status was not associated with willingness to enforce the STP. Nor were school-level factors such as school SES or visibility of smoking associated with the willingness to enforce the STP.

### Interpretation

Our results enrich the initial conceptual framework. The dimension capturing the professional role encompassed two of the three concepts suggested in the realist review: responsibility and confidence (Linnansaari *et al*. 2019). High agreement was found among staff members for the items related to the first factor, which means that, globally, staff members feel that enforcing STPs is one of their professional duties and does not threaten their relationship with students. The second dimension, the sense of shared responsibility, was mainly related to the motivation concept identified in the initial framework (Linnansaari *et al*. 2019). Compared to motivation, which is an individual-level concept, the sharing of rules emphasizes the collective endeavour of STP enforcement and suggests that motivation to enforce the STP depends on how one expects colleagues and parents to behave (Bicchieri 2016, Finch *et al*. 2018). Weaker agreement was found among staff members for the corresponding items. For a significant proportion of the staff, smoking prevention is not a school priority, as suggested to be a prerequisite to achieving the implementation of the STP (Damschroder *et al*. 2009). Furthermore, enforcement as a collective behaviour was not shared: a large proportion felt that they were not supported, either by staff members or by parents. Moreover, the feeling that the students will circumvent the rules anyway also reflects a lack of support on their part. According to May and Finch, to be embedded in a school’s routine, STP enforcement must be bought into by a majority of actors (May and Finch 2009).

Findings relating to the predictors of enforcement strengthen the second dimension identified in the EFA. Believing that colleagues are most often enforcing the rules, and that a majority of them are doing so in a cohesive way, fosters willingness to enforce the STP. This is consistent with social norm theory (Bicchieri 2016): people are more willing to act when they know that others around them are also doing so. The sense that the rules were justified also predicted greater willingness to enforce the STP. So, staff are more willing to enforce rules they perceive as justified, which is consistent with previous assertions that staff will engage in health promotion interventions as long as they do not perceive them to be conflicting with the aims of education (Tjomsland *et al*. 2009, Fielding 2022). We did not find a predictive value of staff members’ smoking status on enforcement, except for daily smokers who feel less responsible for STP enforcement and feel that they have less legitimacy to enforce STPs. Although staff members who smoke are less supportive of smoke-free schools than non-smokers, studies have shown that smokers still express support for measures that target minors as long as they are not targeted themselves (Schumann *et al*. 2006, Bonevski *et al*. 2011, Chung-Hall *et al*. 2018).

School-level factors such as smoking prevalence and visibility of smoking seem to be less relevant when assessing willingness enforce the STP. A previous study showed that greater willingness to enforce STPs was predicted by a higher school socioeconomic status (Mélard *et al*. 2025). Schreuders *et al.* showed that staff are more reluctant to apply stricter rules to adolescents who are already more vulnerable and whose overall situation deprioritizes the smoking issue (Schreuders *et al*. 2020).

### Strengths and limitations

To our knowledge, this is the first study to quantify and analyse the willingness of school staff to enforce STPs using a large sample of staff from different schools in an area where smoking remains prevalent. The study’s main limitation is its geographical reach, as we conducted the survey in a specific Belgian province with high deprivation and frequent smoking. Further studies could test the developed scale in other countries and regions to assess its generalizability and compare the results. Only two dimensions were captured from the scale, however, so a shorter version, with fewer items, could also be tested. The interpretation of the results may also be limited by a reporting bias, for example, non-smoking is reported due to its social desirability. The prevalence of smoking in our sample, however, is similar to that observed among individuals with comparable educational levels in the same province (Gisle and Demarest 2025). Another limitation concerns the variation in the response rate between schools (ranging from 1 to 55%). Nevertheless, supplementary analyses did not reveal any associations between schools’ response rates and schools’ SES, students’ or staff’s smoking prevalence, smoking visibility, gender proportion, or mean age of school staff (Additional file 4). These variables, therefore, do not indicate selection biases.

### Implications

Our study adds to the recent qualitative research by pinpointing the main perceptions held by staff members that schools should address when aiming to become smoke-free. Whereas previous studies have argued that staff members would not feel they had legitimacy to enforce the STP or that they would fear ruining their relationship with the students by doing so, our study showed that these concerns are overstated (Schreuders *et al*. 2019, 2020, Linnansaari *et al*. 2022). Our results pointed more to the need, highlighted in recent studies, for shared responsibility for enforcement: perceived collegial and managerial support, garnering support from the parents, and developing the common view that adolescent smoking is more important than it is currently considered (Linnansaari *et al*. 2019, Schreuders *et al*. 2019, Linnansaari *et al*. 2022). More broadly, our study aligns with qualitative findings from a Danish study, which highlighted that facilitators of STP implementation include the perception of health promotion as a school role and duty and that changes can be achieved by developing a common understanding of the STP (Hjort *et al*. 2021). The authors also identified barriers such as feeling that students have other, more important problems than smoking, and that implementing the STP could influence staff–student relationships. The latter did not emerge in our study.

Harnessing support from the whole school community in the process of enforcing policies, and thereby developing a joint understanding, has already been suggested by others as a way to improve the adoption of smoking bans (Durlak and DuPre 2008, Rozema *et al*. 2016). When people know that they are not acting alone, they are more willing to engage in a specific behaviour (Bicchieri 2016). If stakeholders feel they have ownership of the STP, they will be more likely to support and accept the rules (Kreuter *et al*. 2000). According to the social norm theory and the ACF, changing factual beliefs about STP enforcement and involving the school’s stakeholders in the decision-making process are essential steps to encourage staff members to engage in STP enforcement tasks and feel supported in doing so (Kreuter *et al*. 2000, Bicchieri 2016, Sabatier and Weible 2019, Schreuders *et al*. 2019). Sharing ways of achieving this is a stepping stone to STPs becoming part of the new normative framework (Bicchieri 2016).

Individual schools and educational authorities can use the scale to monitor the current situation and plan activities to enhance school staff’s enforcement skills. By conducting repeated surveys, for example, every 3 years, they can track whether the schools are progressing in the desired direction.

## Conclusion

This study identifies the main arguments that explain poor enforcement of STPs by school staff. The results show that staff members felt that enforcing school rules regarding smoking and vaping was part of their professional role and that they could enforce those rules while staying close to their students, but that they did not have a shared understanding of the rules. Staff members need to feel supported by their colleagues to enforce STPs. Future school smoking prevention interventions could rely on this validated scale to integrate staff members’ positions on the matter and thereby increase the success rate of the intervention.

## Supplementary Material

daag031_Supplementary_Data

## Data Availability

The datasets generated and/or analysed during the current study are not publicly available.
