# Supplementary material for: Why school tobacco bans fail: staff engagement in enforcement in Belgian schools
Source: Health Promot Int. 2026 Mar 3;41(2):daag031. doi: 10.1093/heapro/daag031 (PMC13016720; doi:10.1093/heapro/daag031)
Supplement: daag031_Supplementary_Data [file daag031_supplementary_data.zip › Additional file 2. Evolution scale.docx]

**Evolution of the scale building**

Changes are highlighted in yellow

26/08/2024

How do you see your role in the enforcement of STPs in your school?

| Framework | Previous questions |
| --- | --- |
| Mechanism 1 – Responsibility is part of professional role and duties | It is my responsibility to enforce these rules  I feel I have legitimacy to enforce these rules  It is important to prevent students from smoking |
| Mechanism 2 – Staff contribution to positive outcomes to motivate enforcement | I feel supported enough to enforce these rules |
| Mechanism 3 – Staff’s ability to enforce the rules to be confident to enforce the STP | I know these rules well  I risk damaging my relationship with my students if I enforce these rules |

23/09/2024

Do you agree with the following statements?

| Framework | Previous questions |
| --- | --- |
| Mechanism 1 – Responsibility is part of professional role and duties | It is my responsibility to enforce these rules  I feel I have legitimacy to enforce these rules  It is important to prevent students from smoking |
| Mechanism 2 – Staff contribution to positive outcomes to motivate enforcement | I feel supported enough to enforce these rules |
| Mechanism 3 – Staff’s ability to enforce the rules to be confident to enforce the STP | I know these rules well  I risk damaging my relationship with my students if I enforce these rules |

02/10/2024

Following statements concern the STP of your school. Do you agree with them?

| Framework | Previous questions |
| --- | --- |
| Mechanism 1 – Responsibility is part of professional role and duties | It is my responsibility to enforce these rules  I feel I have legitimacy to enforce these rules  It is important to prevent students from smoking |
| Mechanism 2 – Staff contribution to positive outcomes to motivate enforcement | I feel supported enough to enforce these rules  These rules prevent students from smoking and vaping  Students always get around these rules |
| Mechanism 3 – Staff’s ability to enforce the rules to be confident to enforce the STP | I know these rules well  I risk damaging my relationship with my students if I enforce these rules |

03 – 15/10/2024

Tested by school staff from a Brussels school. Feedback on clarity and understanding of the questions.

15/10/2024

Following statements concern the STP of your school. Do you agree with them?

| Framework | Previous questions |
| --- | --- |
| Mechanism 1 – Responsibility is part of professional role and duties | It is my responsibility to enforce these rules  I feel I have legitimacy to enforce these rules  Smoking prevention is a priority for my school |
| Mechanism 2 – Staff contribution to positive outcomes to motivate enforcement | I feel supported by my colleagues to enforce these rules  I feel supported by the parents to enforce these rules  These rules prevent students from smoking and vaping  Students always get around these rules |
| Mechanism 3 – Staff’s ability to enforce the rules to be confident to enforce the STP | I know these rules well  I risk damaging my relationship with my students if I enforce these rules  I can enforce these rules while remaining close with my students |

30/10/2024

What is your opinion about your school’s STP

| Framework | Previous questions |
| --- | --- |
| Mechanism 1 – Responsibility is part of professional role and duties | It is my responsibility to enforce these rules  I feel I have legitimacy to enforce these rules  Smoking prevention is a priority for my school |
| Mechanism 2 – Staff contribution to positive outcomes to motivate enforcement | I feel supported by my colleagues to enforce these rules  I feel supported by the parents to enforce these rules  These rules protect students from smoking and vaping  Students always get around these rules |
| Mechanism 3 – Staff’s ability to enforce the rules to be confident to enforce the STP | I know these rules well  I risk damaging my relationship with my students if I enforce these rules  I can enforce these rules while remaining close with my students |

31/10/2024

Recheck by staff members

04/11/2024

Survey sent to participating schools
