# Supplementary material for: Why school tobacco bans fail: staff engagement in enforcement in Belgian schools
Source: Health Promot Int. 2026 Mar 3;41(2):daag031. doi: 10.1093/heapro/daag031 (PMC13016720; doi:10.1093/heapro/daag031)
Supplement: daag031_Supplementary_Data [file daag031_supplementary_data.zip › Additional file 3. Exploratory factor analysis.docx]

**Exploratory factor analysis**


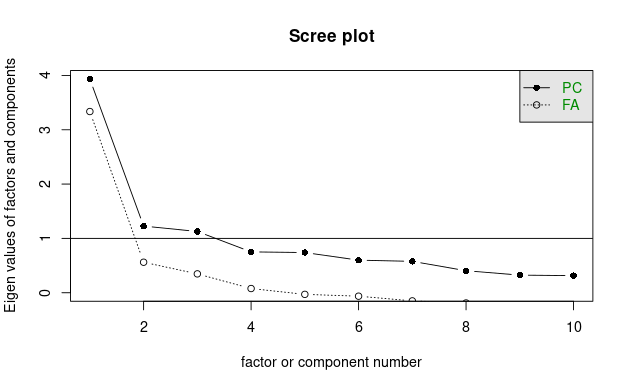


*Figure 1. Scree plot.*


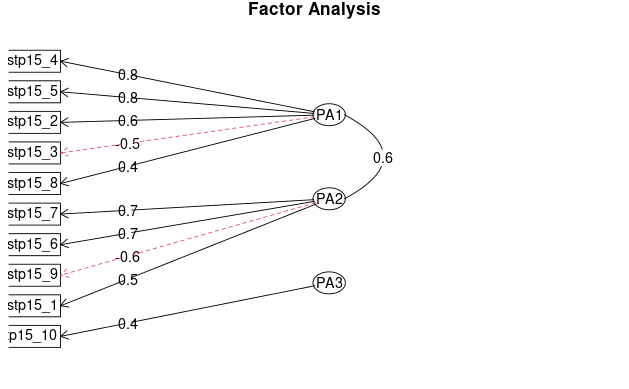


*Figure 2. Three factor solution of the exploratory factor analysis (n = 577), ADHAirE study 2024.*


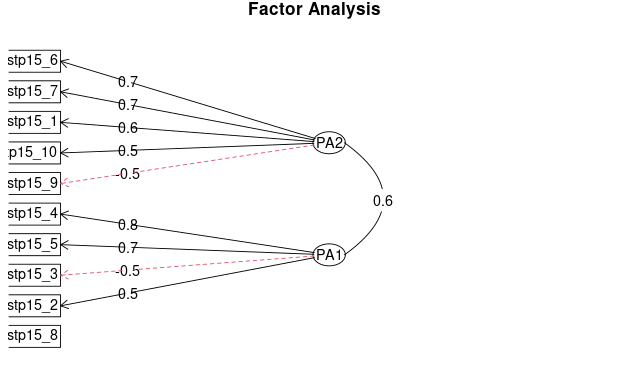


*Figure 3. Two factor solution of the exploratory factor analysis (n = 577), ADHAirE study 2024.*

Table 1. Validity and reliability analysis of the School Smoking Prevention Enforcement Scale – two factor solution of the exploratory factor analysis, (n = 577), ADHAirE study 2024

|  | KMO index | Cronbach’s Alpha | Factor 1 loading | Factor 2 loading | Communalities |
| --- | --- | --- | --- | --- | --- |
| **Overall** | 0.83 | 0.82 | 0.20 | 0.20 | 0.42 |
| **Mechanism 1** |  |  |  |  |  |
| It is my responsibility to enforce these rules | 0.86 | 0.79 | 0.55 | 0.17 | 0.45 |
| I feel legitimate to enforce these rules | 0.83 | 0.78 | 0.75 | 0.09 | 0.66 |
| Smoking prevention is a priority for my school | 0.78 | 0.81 | -0.07 | 0.46 | 0.18 |
| **Mechanism 2** |  |  |  |  |  |
| These rules help protect students from smoking and vaping | 0.84 | 0.80 | -0.06 | 0.60 | 0.31 |
| Students always get around these rules | 0.83 | 0.81 | -0.03 | -0.46 | 0.23 |
| I feel supported by my colleagues to enforce these rules | 0.82 | 0.78 | 0.10 | 0.75 | 0.67 |
| I feel supported by the parents to enforce these rules | 0.82 | 0.79 | -0.03 | 0.75 | 0.53 |
| **Mechanism 3** |  |  |  |  |  |
| I know these rules well | 0.86 | 0.82 | 0.27 | 0.10 | 0.12 |
| I risk damaging the relationship with my students if I enforce these rules | 0.81 | 0.80 | -0.55 | 0.01 | 0.29 |
| I can enforce these rules while remaining close to my students | 0.83 | 0.79 | 0.84 | -0.09 | 0.62 |

Table 2. Validity analysis of the School Smoking Prevention Enforcement Scale – three factor solution of the exploratory factor analysis, (n = 577), ADHAirE study 2024

|  | Factor 1 loading | Factor 2 loading | Factor 3 loading | Communalities |
| --- | --- | --- | --- | --- |
| **Overall** | 0.21 | 0.19 | 0.05 | 0.46 |
| **Mechanism 1** |  |  |  |  |
| It is my responsibility to enforce these rules | 0.62 | 0.07 | 0.23 | 0.52 |
| I feel legitimate to enforce these rules | 0.77 | 0.06 | 0.09 | 0.66 |
| Smoking prevention is a priority for my school | 0.00 | 0.35 | 0.40 | 0.30 |
| **Mechanism 2** |  |  |  |  |
| These rules help protect students from smoking and vaping | 0.01 | 0.51 | 0.21 | 0.33 |
| Students always get around these rules | 0.06 | -0.64 | 0.29 | 0.43 |
| I feel supported by my colleagues to enforce these rules | 0.15 | 0.70 | 0.07 | 0.66 |
| I feel supported by the parents to enforce these rules | 0.03 | 0.70 | 0.04 | 0.52 |
| **Mechanism 3** |  |  |  |  |
| I know these rules well | 0.34 | -0.01 | 0.25 | 0.19 |
| I risk damaging the relationship with my students if I enforce these rules | -0.52 | -0.10 | 0.31 | 0.41 |
| I can enforce these rules while remaining close to my students | 0.79 | -0.02 | -0.13 | 0.61 |
