# Supplementary material for: Why school tobacco bans fail: staff engagement in enforcement in Belgian schools
Source: Health Promot Int. 2026 Mar 3;41(2):daag031. doi: 10.1093/heapro/daag031 (PMC13016720; doi:10.1093/heapro/daag031)
Supplement: daag031_Supplementary_Data [file daag031_supplementary_data.zip › Additional file 4. Response rate analysis.docx]

**Response rate analysis**

Table 1. School staff response rate analysis by school, linear regression analysis, ADHAIRE study 2024

|  | Coefficient (SE) | P value |
| --- | --- | --- |
| Mean age | 1.01 (1.59) | 0.54 |
| Proportion of male | -35.48 (25.14) | 0.18 |
| Prevalence of current smokers in staff | 30.88 (55.86) | 0.59 |
| Visibility of staff smoking inside | -9.21 (19.51) | 0.64 |
| Prevalence of smoking initiation among students | 10.92 (28.21) | 0.70 |
| School SES | 0.78 (0.84) | 0.37 |
